# Brazilian guidelines for the management of brain-dead potential organ donors. The task force of the *Associação de Medicina Intensiva Brasileira, Associação Brasileira de Transplantes de Órgãos*, Brazilian Research in Critical Care Network, and the General Coordination of the National Transplant System

**DOI:** 10.5935/0103-507X.20210001

**Published:** 2021

**Authors:** Glauco Adrieno Westphal, Caroline Cabral Robinson, Alexandre Biasi Cavalcanti, Anderson Ricardo Roman Gonçalves, Cátia Moreira Guterres, Cassiano Teixeira, Cinara Stein, Cristiano Augusto Franke, Daiana Barbosa da Silva, Daniela Ferreira Salomão Pontes, Diego Silva Leite Nunes, Edson Abdala, Felipe Dal-Pizzol, Fernando Augusto Bozza, Flávia Ribeiro Machado, Joel de Andrade, Luciane Nascimento Cruz, Luciano César Pontes Azevedo, Miriam Cristine Vahl Machado, Regis Goulart Rosa, Roberto Ceratti Manfro, Rosana Reis Nothen, Suzana Margareth Lobo, Tatiana Helena Rech, Thiago Costa Lisboa, Verônica Colpani, Maicon Falavigna

**Affiliations:** 1 Hospital Moinhos de Vento - Porto Alegre (RS), Brazil.; 2 Hospital Municipal São José - Joinville (SC), Brazil.; 3 Centro Hospitalar Unimed - Joinville (SC), Brazil.; 4 HCor-Hospital do Coração - São Paulo (SP), Brazil.; 5 Universidade da Região de Joinville - Joinville (SC), Brazil.; 6 Clínica de Nefrologia de Joinville - Joinville (SC), Brazil.; 7 Hospital de Clínicas de Porto Alegre, Universidade Federal do Rio Grande do Sul - Porto Alegre (RS), Brazil.; 8 Universidade Federal de Ciências da Saúde de Porto Alegre - Porto Alegre (RS), Brazil.; 9 Hospital de Pronto de Socorro - Porto Alegre, RS, Brazil.; 10 Coordenação Geral do Sistema Nacional de Transplante - Brasília (DF), Brasil.; 11 Faculdade de Medicina, Universidade de São Paulo - São Paulo (SP), Brazil.; 12 Universidade do Estado de Santa Catarina - Criciúma (SC), Brazil.; 13 Hospital São José - Criciúma (SC), Brazil.; 14 Instituto Nacional de Doenças Infeciosas Evandro Chagas, Fundação Oswaldo Cruz - Rio de Janeiro (RJ), Brazil.; 15 Instituto D’Or de Pesquisa e Educação - Rio de Janeiro (RJ), Brasil.; 16 Discipline of Anesthesiology, Pain and Intensive Care, Hospital São Paulo, Universidade Federal de São Paulo - São Paulo (SP), Brazil.; 17 Organização de Procura de Órgãos de Santa Catarina - Florianópolis (SC), Brazil.; 18 Hospital Sírio-Libanês - São Paulo (SP), Brazil.; 19 Universidade Federal do Rio Grande do Sul - Porto Alegre (RS), Brazil.; 20 Faculdade de Medicina de São José do Rio Preto - São José do Rio Preto (SP), Brazil.; 21 Instituto Nacional de Avaliação de Tecnologias da Saúde, Universidade Federal do Rio Grande do Sul - Porto Alegre (RS), Brazil.; 22 Department of Health Research Methods, Evidence, and Impact, McMaster University - Hamilton, Canada.

**Keywords:** Guidelines, Organ donation, Intensive care, Brain death, GRADE, Diretrizes, Doação de órgãos, Terapia intensiva, Morte encefálica, GRADE

## Abstract

**Objective:**

To contribute to updating the recommendations for brain-dead potential organ donor management.

**Methods:**

A group of 27 experts, including intensivists, transplant coordinators, transplant surgeons, and epidemiologists, answered questions related to the following topics were divided into mechanical ventilation, hemodynamics, endocrine-metabolic management, infection, body temperature, blood transfusion, and checklists use. The outcomes considered were cardiac arrests, number of organs removed or transplanted as well as function / survival of transplanted organs. The quality of evidence of the recommendations was assessed using the Grading of Recommendations Assessment, Development, and Evaluation system to classify the recommendations.

**Results:**

A total of 19 recommendations were drawn from the expert panel. Of these, 7 were classified as strong, 11 as weak and 1 was considered a good clinical practice.

**Conclusion:**

Despite the agreement among panel members on most recommendations, the grade of recommendation was mostly weak.

## Abstract (pt)

**Objetivo:**

Fornecer recomendações para nortear o manejo clínico do potencial doador em morte encefálica.

**Métodos:**

O presente documento foi formulado em dois painéis compostos por uma força tarefa integrada por 27 especialistas de diferentes áreas que responderam a questões dirigidas aos seguintes temas: ventilação mecânica, hemodinâmica, suporte endócrino-metabólico, infecção, temperatura corporal, transfusão sanguínea, e uso de checklists. Os desfechos considerados foram: parada cardíaca, número de órgãos retirados ou transplantados e função/sobrevida dos órgãos transplantados. A qualidade das evidências das recomendações foi avaliada pelo sistema *Grading of Recommendations Assessment, Development, and Evaluation.*

**Resultados:**

Foram geradas 19 recomendações a partir do painel de especialistas. Dessas, 7 foram classificadas como fortes, 11 fracas e uma foi considerada boa prática clínica.

**Conclusão:**

Apesar da concordância entre os membros do painel em relação à maior parte das recomendações, o grau de recomendação é fraco em sua maioria.

## INTRODUCTION

The progress of the process of organ donation for transplantation is essential to increase the deceased-donor pool and to decrease the growing disparity between the number of patients on transplant waiting lists and the availability of organs.^(1,2)^ This process includes the identification of the potential donor, diagnosis of brain death, family support and interview, evaluation of donor eligibility criteria, clinical management of the potential organ donor, and organ procurement and distribution.^(2,3)^ Given the marked clinical instability that occurs in patients who progress to brain death, the application of potential-donor management strategies is crucial to avoid loss of organs due to hypoperfusion or loss of donors due to cardiac arrest.^(1,2,4,5)^

The recommendations presented in this guideline intend to promote a general approach to mitigate the disparity between supply and demand of organs for transplantation.

## OBJECTIVE

To provide recommendations to guide the clinical management of brain-dead potential organ donors aiming to reduce the rate of cardiac arrest of the potential donor and to improve organ viability for transplantation.

## METHODS

The present document provides a partial update on the 2011 Brazilian Guidelines for Management of Adult Potential Multiple-Organ Deceased Donors.^(6-8)^ The target audience of this guideline is health care professionals, especially physicians and nursing staff working in adult intensive care units (ICUs) and emergency departments, who are involved in the care of adult individuals with known or suspected brain death.

The clinical issues addressed by the guideline were divided into the following major topics: (1) ventilatory support; (2) hemodynamic support; (3) endocrine, metabolic and nutritional management; (4) specific aspects that include infection and sepsis, red blood cell transfusion, and body temperature control; and (5) goal-directed therapy. For each clinical issue, operational questions were developed and framed using the population-intervention-comparison-outcome (PICO) format. The population of interest consists of potential organ donors with known or suspected brain death,^(3)^ hereafter referred to as potential donors. The outcomes considered for decision-making were cardiac arrest, the number of organs recovered or transplanted per donor, and graft function or graft survival.

For each clinical issue, rapid systematic reviews^(9,10)^ were conducted using the following search strategy: (1) Review of the reference lists of Brazilian guidelines^(6-8)^ and the Society of Critical Care Medicine (SCCM)^(11)^ statement on the management of the potential organ donor; (2) Review of related topics in the DynaMed and UpToDate databases; and (3) PubMed search focusing on systematic reviews and clinical trials published until October 2016 and until January 2017. Quality of evidence was assessed using the Grading of Recommendations Assessment, Development, and Evaluation (GRADE) system.^(12)^

The recommendations were prepared and submitted to 2 face-to-face expert panels held in November 2016, and February 2017. For each recommendation, the direction of the course of action was discussed (whether to perform or not to perform the proposed action), and the strength of the recommendation was classified as strong or weak according to the GRADE system.^(12)^ After the last panel meeting, a new systematic search covering the period from October 2016 to May 2020 was carried out to identify new evidence that could potentially modify the recommendations. From June to July 2020, a Delphi process was performed with the panelists to present the results of the literature update and review the direction and strength of the recommendations.

## RESULTS

**Figure 1:**
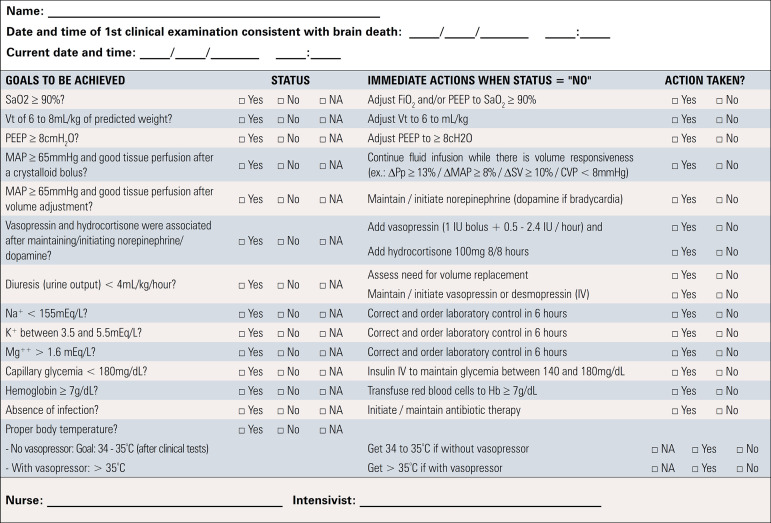
Evidence-based bed-side checklist for clinical management of brain-dead potential organ donors.

**Figure 2:**
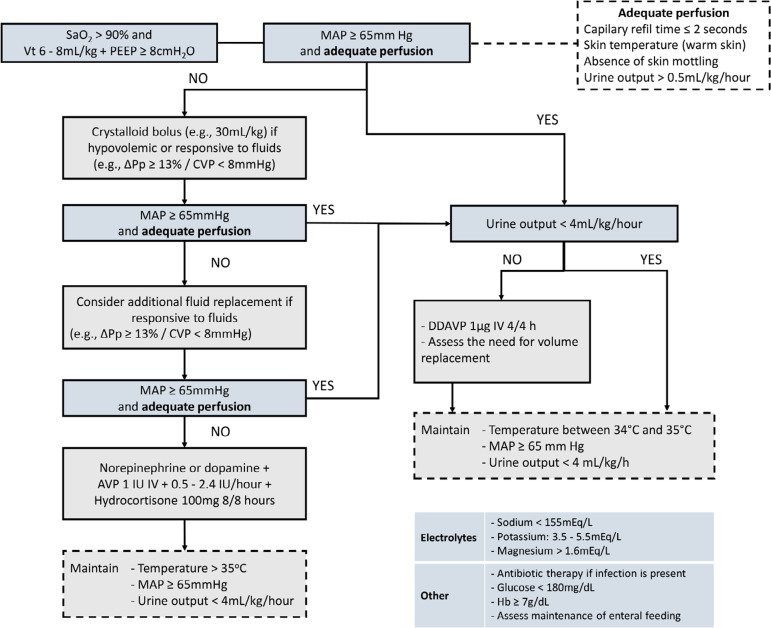
Fluxograma para manutenção clínica do potencial doador de órgãos em morte encefálica.

A total of 19 recommendations were drawn from the expert panel. Of these, 7 were classified as strong, 11 as weak, and 1 was considered as good clinical practice. Table 1 shows a summary of the recommendations and figure 1 presents the checklist based on the main recommendations to assist in bedside monitoring of clinical goals related to the recommendations and in the application of the management strategies. Figure 2 depicts graphically the flow of the recommendations along the clinical management.

**Table 1:** Summary of recommendations

Recommendations	Level of evidence	Grade of recommendation	Practical considerations
**Ventilatory support**			
1. We recommend using a lung-protective ventilation strategy in all PDs.	Low	Strong	Vt between 6 and 8mL/kg of predicted body weight and PEEP of 8 to 10cmH_2_O.
			Adjust FiO_2_ and PEEP to obtain SaO_2_ > 90%.
			Perform apnea testing with CPAP.
2. We suggest not using ARM routinely in PDs.	Very low	Weak	ARM can be considered if there is refractory hypoxemia in hemodynamically stable PDs.
**Hemodynamic support**			
3. We recommend performing initial volemic expansion in hemodynamically unstable PDs with hypovolemia or responsive to fluids according to fluid responsiveness assessment.		Good clinical practice	Initial volume expansion with 30mL/kg of crystalloids.
			Assess fluid status and responsiveness for additional fluid replacement.
			Preferably use dynamic parameters.
			Neutral or negative fluid balance after achieving hemodynamic stability.
4. We recommend administering norepinephrine or dopamine to control blood pressure in PDs who remain hypotensive after volemic expansion.	Very low	Strong	Start adrenergic vasopressors to obtain a MAP ≥ 65mmHg. Dopamine is the vasopressor of choice when there is bradycardia. Consider the potential arrhythmogenic effect of dopamine, which implies the risk of PD loss due to cardiac arrest.
5. We suggest not using low-dose dopamine for renal protection in PDs.	Very low	Weak	Consider the potential arrhythmogenic effect of dopamine, which implies the risk of PD loss due to cardiac arrest.
**Endocrine, electrolyte and nutritional management**			
6. We recommend combining AVP in PDs receiving norepinephrine or dopamine.	Low	Strong	Combine AVP (1 IU bolus + 0.5 - 2.4 IU/h) with norepinephrine or dopamine.
7. We recommend administering AVP or DDAVP to control polyuria in PDs with diabetes insipidus.	Low	Strong	AVP if vasopressors are required.
			DDAVP (1 - 2µg IV 2 to 4 hours) if vasopressors are not required.
8. We suggest combining low-dose corticosteroids in PDs receiving norepinephrine or dopamine.	Low	Weak	Combine 300mg IV/day in PDs with norepinephrine or dopamine.
9. We suggest not using thyroid hormones routinely in PDs.	Very low	Weak	There are no hemodynamic benefits.
			They can be considered if prolonged management is required.
10. We suggest performing glycemic control in PDs.	Very low	Weak	Administer insulin to achieve a glucose level of 140 to 180mg/dL.
			Monitor blood glucose at least every 6 hours.
11. We suggest maintaining serum sodium levels <155mEq/dL in PDs.	Very low	Weak	Correct water deficit with hypotonic fluids.
			Correct hypovolemia.
12. We recommend maintaining serum potassium levels between 3.5 and 5.5mEq/L in PDs.	Very low	Strong	
13. We recommend maintaining serum magnesium levels > 1.6mEq/L in PDs.	Very low	Strong	
**Other aspects**			
14. We suggest maintaining nutritional support in PDs if well tolerated.	Very low	Weak	
15. We recommend using antibiotics in PDs with infection or sepsis.	Low	Strong	Maintain appropriate antibiotic therapy in the donor for at least 24 hours.
			Collect cultures from different sites in all donors.
16. We suggest maintaining body temperature above 35oC in hemodynamically unstable PDs.	Very low	Weak	Monitor core temperature.
			Prevent and treat hypothermia in PDs receiving vasoactive amines.
17. We suggest inducing hypothermia (34 - 35oC) in PDs without hemodynamic instability.	Low	Weak	Monitor core temperature.
			Induce hypothermia by applying ice packs in PDs not receiving vasoactive amines.
18. We suggest transfusing packed red blood cells in PDs with hemoglobin levels < 7g/dL.	Very low	Weak	
19. We suggest using goal-directed protocols during the management of PDs.	Very low	Weak	Monitor care using evidence-based clinical goal-directed checklists.

PD - potential donor; Vt - total volume; PEEP - positive-end expiratory pressure; FiO_2_ - fraction of inspired oxygen; SaO_2_ - arterial oxygen saturation; CPAP - continuous positive airway pressure; ARM - alveolar recruitment maneuver; MAP - mean arterial pressure; AVP - arginine-vasopressin; DDAVP - 1-deamino-8-D-arginine-vasopressin; IV - intravenous.

SaO_2_ - arterial oxygen saturation; FiO_2_ - fraction of inspired oxygen; PEEP - positive end-expiratory pressure; Vt - tidal volume; MAP - mean arterial pressure; ΔPp - pulse pressure respiratory variation; ΔMAP - mean arterial pressure variation; ΔSV - stroke volume respiratory variation; CVP - central venous pressure; Na+ - sodium; K+ - potassium; Mg++ -magnesium; Hb - hemoglobin; NA - not available/not applicable.

SaO_2_ - arterial oxygen saturation; Vt - total volume; MAP - mean arterial pressure; ∆Pp - pulse pressure respiratory variation; CVP - central venous pressure; AVP - arginine-vasopressin; DDAVP - 1-deamino-8-D-arginine-vasopressin; IV - intravenous.

### Ventilatory support recommendations

Two recommendations directed to ventilatory care (recommendations 1 and 2 in Table 1) were generated, with emphasis on the use of the protective ventilation strategy, which consists of the association of tidal volume of 6 to 8mL/kg and positive end-expiratory pressure (PEEP) of 8 to 10cmH_2_O in potential donors with normal lungs, in addition to the suggestion not using alveolar recruitment maneuvers routinely. As practical considerations, we added the possibility of associating the continuous positive pressure apnea test (CPAP) to the protective strategy, to avoid hypoxemia during the test, as well as the fractional inspired oxygen (FiO_2_) and PEEP titration aiming at an arterial oxygen saturation (SaO_2_) > 90% to favor the oxygenation of tissues.^(13-21)^

### Hemodynamic support recommendations

Three recommendations were generated about hemodynamic care (recommendations 3 to 5 in Table 1). It is suggested the use of 30mL/kg crystalloid aliquots in potential donors who are hypotensive (mean arterial pressure - MAP < 65mmHg) and with signs of fluid-responsiveness (preferably measured with dynamic parameters), in order to mitigate the occurrence of volume overload.^(22-33)^

If the pressure goal of MAP ≥ 65mmHg is not achieved with the initial volume expansion, immediate norepinephrine infusion should be started to achieve this target.^(34-37)^ The use of dopamine can be considered in cases of bradycardia with signs of low cardiac output,^(6,38,39)^ but its potential of dopamine should be considered.^(40)^ The administration of low doses of dopamine is not recommended, as the survival benefits of renal and cardiac grafts are not clear and as its potential arrhythmogenic effect increases the risks of cardiac arrest.^(41-44)^

### Endocrine and electrolyte management recommendations

**Endocrine management -** In table 1 there are five recommendations regarding endocrine management, referring to the use of arginine-vasopressin (AVP), hydrocortisone, desmopressin (DDAVP), thyroid hormones and insulin (recommendations 6, 7, 8, 9 and 10 in Table 1, respectively). The administration of AVP (initial bolus of 1 IU followed by the infusion of 0.5 IU/hour to 2.4 IU/hour) and hydrocortisone (100mg intravenous every 8 hours) in potential donors using norepinephrine or dopamine decreases the requirement for adrenergic vasopressors, is associated with a lower incidence of cardiovascular deterioration and contributes to the control of polyuria when diabetes insipidus is present.^(45-55)^ Arginine-vasopressin and hydrocortisone should be started at the same time as the adrenergic vasopressor infusion begins. Desmopressin is indicated to control polyuria (diuresis > 4mL/kg/hour) in potential donors with diabetes insipidus who maintain adequate blood pressure without adrenergic vasopressors. Arginine-vasopressin and DDAVP can be associated in refractory cases.^(56,57)^ Although the intranasal route is possible, the preferred route is intravenous, in doses of 1 - 2µg every 2 to 4 hours,^(8,11,13)^ until a diuresis < 4mL/kg/hour is obtained.^(56-59)^ Although brain death is associated with a drop in thyroid hormone levels, there is no evidence to justify its use in the potential donor, even in potential donors with hemodynamic instability or impaired cardiac function.^(60-70)^ Finally, considering the potential benefit of glycemic control over renal function, it is suggested to keep the blood glucose of potential donors between 140 to 180mg/dL with administration of subcutaneous or intravenous insulin.^(71-79)^

**Electrolytic management -** Three recommendations were generated regarding electrolytic control in the potential donor (recommendations 11, 12 and 13 in Table 1). Hypernatremia in the potential donor is often associated with hypovolemia, and should be controlled with volume expansion, replacement of hypotonic solutions and polyuria control with AVP or DDAVP, in addition to monitoring serum sodium for levels < 155mg/dL.^(11,80-86)^ Changes in potassium and magnesium levels are also common and are related to cardiac arrhythmias. It is suggested to monitor the levels of these electrolytes and institute corrective measures, aiming at serum levels of potassium between 3.5 and 5.5mEq/L and of magnesium above 1.6mEq/L.^(87-93)^

### Other aspects of potential donor management

**Nutritional support -** It is suggested that the nutritional supply of the potential donor be continued if there are no contraindications (recommendation 14 in Table 1), due to the potential benefits on intestinal mucosal trophism and increased hepatic glycogen stores.^(7,9,57)^ In individuals who have already been receiving full nutritional support, the calorie intake should be reduced by 15% to 30%, in addition to considering a minimum caloric supply (eg 500kcal) in potential donors who have not been receiving enteral diet before diagnosis of brain death.^(7,9,57,94-97)^

**Infection and sepsis -** The risk of transmission of bacterial infection between organ donors and recipients is low and the infection in the donor does not appear to compromise the outcomes. It is recommended to use antibiotics in the potential donors who present infection or sepsis (recommendation 15 in Table 1). The risks of infection transmission are lower with appropriate antibiotic therapy in the potential donor for at least 24 hours, followed by maintenance of the antibiotic in the recipient for 7 to 14 days.^(98-107)^ In addition, cultures of all potential donors should be collected from different sites, as well as antibiotics administered to recipients, preferably guided by cultures.^(100,108-111)^

**Control of body temperature -** Two recommendations were generated regarding the control of body temperature (recommendations 16 and 17 in Table 1). In the presence of hemodynamic instability, it is suggested to keep the potential donor in normothermia (> 35^o^C) to reduce the risk of arrhythmias, cardiovascular dysfunction and cardiac arrest. On the other hand, among potential donors who are hemodynamically stable, the induction of moderate hypothermia (34 - 35^o^C) has been associated with better renal graft function, however this procedure requires monitoring of central temperature, which is not always available in all ICUs.^(112-116)^

**Red blood cell transfusion** *-* Anemia can compromise the delivery of oxygen to the organs that are intended to be preserved for transplantation. As we do not know the hemoglobin levels necessary to contribute to the adequate transport of oxygen in potential donors, it is suggested to transfuse red blood cells when the hemoglobin is less than 7g/dL, according to the usual practice in other critical patients (recommendation 18 of the Table 1).^(117)^

**Goal-guided protocols -** The adoption of goal-directed checklists to guide the maintenance of potential donor can contribute to the increase in the number of donated organs, influence the function of the graft and decrease losses of potential donors due to cardiac arrest. In general, the outcomes are associated with the number of goals achieved during the maintenance of the potential donor, which includes ventilatory, hemodynamic and endocrine-metabolic management goals.^(24,28,29,79,118-127)^ Therefore, it is suggested to use goal-guided protocols during the management of potential donors.

## DISCUSSION

The present guideline aimed to provide parameters to optimize the clinical management of potential donors based on the available evidence, aiming to improve the quality of organs for transplantation and to reduce donor losses.

This guideline evaluated a broad volume of treatments and we performed rigorous PICO-driven research to provide the recommendations based on standardized rapid review methods.^(9,10)^ Potential limitations are the low or very low certainty in the evidence identified for many of the questions, and indirect evidence that did not change after the systematic review update. However, management recommendations are consistent with similar documents recently published.^(11,128,129)^

Some observational studies have reported that the application of a checklist to guide the management of brain-dead potential donors may help reduce the rate of cardiac arrest in potential donors and increase the number of organs recovered per donor.^(24,79,120,122,123,125,127,130,131)^ In this context, we used the main recommendations of the present guideline to develop an evidence-based clinical goal-directed checklist (Figure 1) with the purpose of providing transplant coordinators and ICU professionals with essential information to optimize the care of potential donors.

However, because the available studies highlighting the role of potential donor management checklists are observational, there is insufficient evidence to support the systematic use of checklists in the management of potential donors. Therefore, we proposed the Donation Network to Optimize Organ Recovery Study (DONORS; NCT03179020), which is a parallel cluster randomized controlled multicenter trial that aims to test the effectiveness of the implementation of a checklist containing goals and recommendations of care in reducing organ donor losses due to cardiac arrest and increasing the number of organs recovered per donor.^(132)^ The implementation of the checklist should be preceded by the appropriate training of intensive care teams and transplant coordinators. We suggest applying the checklist at the bedside immediately after the first clinical examination for the diagnosis of brain death, repeating the application, ideally, every 6 hours until organ procurement for transplantation. We also suggest that a member of the transplant coordination office or a designated professional of the ICU or emergency department apply the checklist at the bedside. The same individual will also be responsible for personally prompting the physician in charge to modify the clinical management if any inappropriate aspect of care, according to the checklist, is noted.

## DECLARATIONS

**Availability of data and material**: All relevant data are within the paper and its Supporting Information files.

**Funding**: This guideline was funded by the Brazilian Ministry of Health through the *Programa de Apoio ao Desenvolvimento Institucional do Sistema Único de Saúde* (PROADI-SUS). The funding body has no role in the coordination of the guideline.

**Authors’ contributions**: All authors, except for AR, DFSP, FDP, RCM, and RRN, participated in at least one of the expert panels. All authors read and approved the final manuscript. The detailed contribution of each author is presented in supplementary material.

**Acknowledgments**: The authors thank the Brazilian Ministry of Health and the General *Coordenação Geral do Sistema Nacional de Transplantes* (CGSNT), as well as *Hospital Moinhos de Vento*, the *Associação Brasileira de Transplantes de Órgãos* (ABTO), the *Associação de Medicina Intensiva Brasileira* (AMIB) Committee for Organ Donation for Transplant, and the Brazilian Research in Intensive Care Network (BRICNet) for their support.

**Permission:** The publication of this document in RBTI was previously authorized by Permission dept of the Annals of Intensive Care Journal, according to the following terms:

*Being Annals of Intensive Care a fully Open Access journal using a CC-BY 4.0 license (https://creativecommons.org/licenses/by/4.0/), you can publish and translate in Portuguese the guidelines. The only requirement is that RBTI provides credit to the original article and indicate what changes are made, if any.*

Therefore, we declare that the changes made in this document are limited to the condensation of the theoretical support of the recommendations and the discussion session, maintaining the content of the recommendations in full.
