# Supplementary material for: Brazilian guidelines for the management of brain-dead potential organ donors. The task force of the Associação de Medicina Intensiva Brasileira, Associação Brasileira de Transplantes de Órgãos, Brazilian Research in Critical Care Network, and the General Coordination of the National Transplant System
Source: Rev Bras Ter Intensiva. 2021 Jan-Mar;33(1):1–11. doi: 10.5935/0103-507X.20210001 (PMC8075340; doi:10.5935/0103-507X.20210001)
Supplement: Supplementary file 1 [file rbti-33-01-0001-suppl1.pdf]

Brazilian guidelines for the management of brain-dead potential organ donors. The task force of the *Associação de Medicina Intensiva Brasileira, Associação Brasileira de Transplantes de Órgãos*, Brazilian Research in Critical Care Network, and the General Coordination of the National Transplant System

Diretrizes brasileiras para o manejo de potenciais doadores de órgãos em morte encefálica. Uma força-tarefa composta por Associação de Medicina Intensiva Brasileira, Associação Brasileira de Transplantes de Órgãos, Brazilian Research in Critical Care Network e Coordenação Geral do Sistema Nacional de Transplantes

Glauco Adrieno Westphal[1,2,3], Caroline Cabral Robinson[1], Alexandre Biasi Cavalcanti[4], Anderson Ricardo Roman Gonçalves[5,6], Cátia Moreira Guterres[1], Cassiano Teixeira[7,8], Cinara Stein[1], Cristiano Augusto Franke[7,9], Daiana Barbosa da Silva[1], Daniela Ferreira Salomão Pontes[10], Diego Silva Leite Nunes[10], Edson Abdala[11], Felipe Dal-Pizzol[12,13], Fernando Augusto Bozza[14,15], Flávia Ribeiro Machado[16], Joel de Andrade[17], Luciane Nascimento Cruz[1], Luciano César Pontes Azevedo[18], Miriam Cristine Vahl Machado[3], Regis Goulart Rosa[1], Roberto Ceratti Manfro[7], Rosana Reis Nothen[19], Suzana Margareth Lobo[20], Tatiana Helena Rech[7], Thiago Costa Lisboa[7], Verônica Colpani[1], Maicon Falavigna[1,21,22]

Appendix 1 - Working group and the contributions of each participant

Name	Contribution	Attended the scope meeting (January 26, 2016)	Searched the literature and reviewed evidence	Attended the 1st recommendations meeting (November 3, 2016)	Attended the 2nd recommendations meeting (February 9, 2017)	Reviewed and approved the final document
Alexandre Biasi Cavalcanti<br>Intensivist	Panel member		x	x	x	x
Anderson Ricardo Roman Gonçalves<br>Nephrologist	Technical review					x
Caroline Cabral Robinson<br>Physical therapist	Methodologist	x	x	X	x	x
Cátia Moreira Guterres<br>Pharmacist	Methodologist	x		x	x	x
Cassiano Teixeira<br>Intensivist	Panel member	x	x	x	x	x
Cinara Stein<br>Physical therapist	Methodologist		x	x	x	x
Cristiano Augusto Franke<br>Intensivist	Panel member			x	x	x
Daiana Barbosa da Silva<br>Nurse	Panel member			x	x	x
Daniela Ferreira Salomão Pontes<br>Intensivist	Technical review					x
Diego Silva Leite Nunes<br>Intensivist	Panel member			x		x
Edson Abdala<br>Intensivist	Panel member		x	x		x
Felipe Dal-Pizzol<br>Intensivist	Panel member		x			x
Fernando Augusto Bozza<br>Intensivist	Panel member				x	x
Flávia Ribeiro Machado.<br>Intensivist	Panel member		x	x	x	x
Glauco Adrieno Westphal<br>Intensivist	Panel member<br>Guideline's co-chair	x	x	x	x	x
Joel de Andrade<br>Intensivist	Panel member			x		x
Luciane Nascimento Cruz<br>Psychiatrist	Panel member				x	x

Continue...

...continuation

Luciano César Pontes de Azevedo<br>Intensivist	Panel member		x	x	x	x
Maicon Falavigna<br>Epidemiologist	Methodologist<br>Guideline's co-chair	x	x	x	x	x
Miriam Cristine Vahl Machado<br>Intensivist	Panel member		x	x		x
Regis Goulart Rosa<br>Intensivist	Panel member			x		x
Roberto Ceratti Manfro<br>Nephrologist	Technical review					x
Rosana Reis Nothen<br>Pediatrician	Technical review					x
Suzana Margareth Lobo<br>Intensivist	Panel member		x	x		x
Tatiana Helena Rech<br>Intensivist	Panel member		x	x		x
Thiago Lisboa<br>Intensivist	Panel member			x	x	x
Verônica Coplani<br>Physical therapist	Methodologist		x	x	x	x

Appendix 2 - Conflict of interest statement

Summary table of conflicts of interest declared by the guideline participants

Name	Conflict of interest declared		Decision on participation
	Question	Description of conflict of interest	
Alexandre Biasi Cavalcanti	None	Not applicable	Not applicable
Anderson Ricardo Roman Gonçalves	None	Not applicable	Not applicable
Caroline Cabral Robinson	None	Not applicable	Not applicable
Cátia Moreira Guterres	None	Not applicable	Not applicable
Cassiano Teixeira	None	Not applicable	Not applicable
Cinara Stein	None	Not applicable	Not applicable
Cristiano Augusto Franke	None	Not applicable	Not applicable
Daiana Barbosa da Silva	None	Not applicable	Not applicable
Daniela Ferreira Salomão Pontes	5b	Coordinator of the National Transplant System of the Brazilian Ministry of Health	Did not participate in the panel
Diego Silva Leite Nunes	5b	Health regulation consultant of the General Coordination Office of the National Transplant System of the Brazilian Ministry of Health	Declared and participated
Edson Abdala	None	Not applicable	Not applicable
Felipe Dal-Pizzol	None	Not applicable	Not applicable
Fernando Augusto Bozza	None	Not applicable	Not applicable
Flávia Ribeiro Machado	None	Not applicable	Not applicable
Glauco Adrieno Westphal	None	Not applicable	Not applicable
Joel de Andrade	None	Not applicable	Not applicable
Luciane Nascimento Cruz	None	Not applicable	Not applicable
Luciano César Pontes de Azevedo	None	Not applicable	Not applicable
Maicon Falavigna	None	Not applicable	Not applicable
Miriam Cristine Vahl Machado	None	Not applicable	Not applicable
Regis Goulart Rosa	None	Not applicable	Not applicable
Roberto Ceratti Manfro	5b	President of the Brazilian Association for Organ Transplantation	Did not participate in the panel
Rosana Reis Nothen	5b	Coordinator of the National Transplant System of the Brazilian Ministry of Health	Did not participate in the panel
Suzana Margareth Lobo	None	Not applicable	Not applicable
Tatiana Helena Rech	None	Not applicable	Not applicable
Thiago Costa Lisboa	None	Not applicable	Not applicable
Verônica Coplani	None	Not applicable	Not applicable

CONFLICT OF INTEREST STATEMENT

The declaration of interests was adapted from the World Health Organization's declaration of interest form. All group members must disclose their potential conflicts of interest on the Declaration of Interest Form below. Participants who had any relevant conflicts of interest associated with one or more questions of the document would not be able to participate in the discussion of these specific subjects, but would be allowed full participation in the discussion of all other subjects, including voting if no consensus was reached. One participant declared to have a conflict of interest relevant to question 5b, as shown in the summary table of conflicts of interest declared by the guideline participants.

DECLARATION OF INTEREST FORM*

*Document adapted from the World Health Organization's declaration of interest form.

The Ministry of Health, seeking scientific basis for decisions related to health policies, depends, in many circumstances, on the assistance of external experts who may have interests related to their expertise. To ensure the highest integrity and public confidence in its activities, the Ministry of Health requires that these experts disclose any circumstances that could give rise to a potential conflict of interest related to the subject of the activity in which they will be involved.

Within this context, we would like to invite you to complete the form below, which will help you disclose any financial, professional or other interest that may affect your participation as a member of the drafting group of the **Guidelines for Maintenance of Potential Multiple Organ Deceased Donors.** The form will be submitted to the Guideline's coordinating team.

Name:
Institution:
Email:

Date and title of the meeting or guideline in which you are involved, including description of subject matter to be considered:
Date: ____

____

Please answer each of the questions below. If the answer to any of the questions is "YES", describe the circumstances on the last page of the form.

The term "you" refers to yourself and your immediate family members (i.e., spouse or partner with whom you have a similar close personal relationship, and your children). "Commercial entity" includes any commercial business, an industry association, research institution or other enterprise whose funding is significantly derived from commercial sources with an interest related to the subject of the meeting or of this guideline. "Organization" includes a governmental, international or non-profit organization. "Meeting" includes a series or cycle of meetings.

**1. EMPLOYMENT AND CONSULTING**			
Within the past 4 years, have you received remuneration from a commercial entity or other organization with an interest related to the subject of the meeting or of this guideline?			
1a	Employment	Yes ( )	No ( )
1b	Consulting, including service as a technical or other advisor	Yes ( )	No ( )
**2. RESEARCH SUPPORT**			
Within the past 4 years, have you or has your research unit received support from a commercial entity or other organization with an interest related to the subject of the meeting or of this guideline?			
2a	Research support, including grants, collaborations, sponsorships, and other funding	Yes ( )	No ( )
2b	Non-monetary support valued at more than R$ 1000.00 overall (include equipment, facilities, research assistants, paid travel to meetings, etc.)	Yes ( )	No ( )
	Support (including honoraria) for being on a speakers bureau, giving speeches or training for a commercial entity or other organization with an interest related to the subject of the meeting or of this guideline?		
**3. INVESTMENT INTERESTS**			
Do you have current investments (valued at more than R$ 10 000.00) in a commercial entity with an interest related to the subject of the meeting or of this guideline? Please also include indirect investments, such as a trust or holding company. You may exclude mutual funds, pension funds or similar investments that are broadly diversified and on which you exercise no control.			
3a	Stocks, bonds, other securities	Yes ( )	No ( )
3b	Commercial business interests (e.g., proprietorships, partnerships, joint ventures, board memberships)	Yes ( )	No ( )
**4. INTELLECTUAL PROPERTY**			
Do you have any intellectual property rights that might be enhanced or diminished by the outcome of the meeting or of this guideline?			
4a	Patents, trademarks, or copyrights (including pending applications)	Yes ( )	No ( )
4b	Proprietary know-how in a substance, technology or process	Yes ( )	No ( )
**5. PUBLIC STATEMENTS AND POSITIONS (DURING THE PAST 3 YEARS)**			
5a	As part of a regulatory, legislative or judicial process, have you provided an expert opinion or testimony, related to the subject of the meeting or of this guideline, for a commercial entity or other organization?	Yes ( )	No ( )
5b	Have you held an office or other position, paid or unpaid, where you represented interests or defended a position related to the subject of the meeting or of this guideline?	Yes ( )	No ( )
**6. ADDITIONAL INFORMATION**			
6a	If not already disclosed above, have you worked for the competitor of a product that is the subject of the meeting or of this guideline, or will your participation in the meeting or this guideline enable you to obtain access to a competitor's confidential proprietary infor-mation, or create for you a personal, professional, financial or business competitive ad-vantage?	Yes ( )	No ( )
6b	To your knowledge, would the outcome of the meeting or of this guideline benefit or adversely affect interests of others with whom you have substantial common personal, professional, financial or business interests (such as your adult children or siblings, close professional colleagues, administrative unit or department)?	Yes ( )	No ( )
6c	Excluding the Ministry of Health or Hospital Moinhos de Vento, has any person or entity paid or contributed toward your travel costs related to this meeting or guideline?	Yes ( )	No ( )
6d	Have you received any payments (other than for travel costs) or honoraria for speaking publicly on the subject of this meeting or guideline?	Yes ( )	No ( )
6e	Is there any other present aspects or circumstances not addressed above that might be perceived as affecting your objectivity or independence?	Yes ( )	No ( )

EXPLANATION of "YES" responses: If the answer to any of the above questions is "YES", briefly describe the circumstances on this page (below). If you do not describe the nature of an interest or if you do not provide the amount or value involved, the conflict will be assumed to be significant.

**Numbers 1 - 4**				
Type of interest, question number and category (e.g., intellectual property 4.a copyrights) and basic descriptive details	Name of company, organization, or institution	Belongs to you, a family member, employer, research unit or other?	Amount of income or value of interest (if not disclosed, is assumed to be significant)	Current interest (or year ceased)
**Numbers 5 - 6**<br>Describe the subject, specific circumstances, parties involved, time frame and other relevant details				

CONSENT TO DISCLOSURE

By completing and signing this form, you consent to the disclosure of any relevant conflicts of interest to other meeting participants and in the resulting guideline or product.

DECLARATION

I hereby declare on my honor that the disclosed information is true and complete to the best of my knowledge.

Should there be any change to the above information, I will promptly notify the responsible staff of this guideline and complete a new declaration of interest form that describes the changes. This includes any change that occurs before or during the meeting or the guideline itself and through the period up to the publication of the final results or completion of the activity concerned.

Date: ________________ Signature: ______________________________
